# 达芬奇机器人与电视胸腔镜辅助非小细胞肺癌根治术近期疗效配对的病例对照研究

**DOI:** 10.3779/j.issn.1009-3419.2018.03.18

**Published:** 2018-03-20

**Authors:** 锋 代, 世广 许, 惟 徐, 仁泉 丁, 博 刘, 浩 孟, 云腾 康, 祥瑞 孟, 杰 林, 述民 王

**Affiliations:** 110015 沈阳，中国人民解放军沈阳军区总医院胸外科 Department of Thoracic Surgery, General Hospital of Shenyang Military, Shenyang 110015, China

**Keywords:** 达芬奇机器人手术, 电视胸腔镜手术, 肺肿瘤, 近期疗效, 配对研究, Robot-assisted thoracic surgery, Video-assisted thoracic surgery, Lung neoplsms, Short-term outcomes, Matching case-control study

## Abstract

**背景与目的:**

作为20世纪最伟大发明之一的达芬奇机器人手术系统代表了精准微创外科的发展方向，本文旨在比较达芬奇机器人与电视胸腔镜辅助肺癌根治术的近期疗效。

**方法:**

回顾性分析自2014年1月-2017年1月在我院行达芬奇机器人手术以及行电视胸腔镜辅助手术治疗非小细胞肺癌患者的相关临床资料，两组各纳入45例并进行配对的病例对照研究。比较两组的手术时间、术中失血量、术中淋巴结清扫数量、淋巴结清扫站数、术后带管时间、术后住院天数、术后第1日胸引液的量以及术后胸引液的总量。

**结果:**

两组均无中转开胸及围术期死亡病例。机器人组与胸腔镜组的术中失血量[（50.30±32.33）mL *vs*（208.60±132.63）mL]与术后第1日胸引液的量[（275.00±145.42）mL *vs*（347.60±125.80）mL]比较，机器人组均少于胸腔镜组；机器人组与胸腔镜组的淋巴结清扫数量[（22.67±9.67）个*vs*（15.51±5.41）个]及淋巴结清扫站数[（6.31±1.43）站*vs*（4.91±1.04）站]比较，机器人组均多于胸腔镜组，差异具有统计学意义；两组的其他指标比较，差异均无统计学意义。

**结论:**

达芬奇机器人行非小细胞肺癌根治术安全、有效，在近期术后疗效上优于胸腔镜组。

## Abstract (en)

**Background and objective:**

Da Vinci Surgical System is one of the greatest inventions of the 20th century, which represents the development direction of the precise minimally invasive surgical techniques, the aim of this study was to comparing the short-term outcomes between da Vinci robot-assisted lobectomy and video-assisted thoracic surgery (VATS) lobectomy for non-small cell lung cancer.

**Methods:**

45 pairs of non-small cell lung cancer patients underwent pulmonary lobectomy with da Vinci Robotic assisted thoracoscopic (RATS) and VATS approach during the same period from January 2014 to January 2017. The operative time, estimated blood loss (EBL), total number and total groups of dissected lymph nodes, postoperative duration of drainage, the first day volume of drainage, total volume of drainage were compared.

**Results:**

No perioperative death and convertion to thoracotomy occured in both groups. There were significant difference between RATS group and VATS group in EBL [(50.30±32.33) mL *vs* (208.60±132.63) mL], the first day volume of drainage [(275.00±145.42) mL *vs* (347.60±125.80) mL], the dissected total number [(22.67±9.67) *vs* (15.51±5.41)] and total team [(6.31±1.43) *vs* (4.91±1.04)] of lymph node. There were no significant difference in other outcomes.

**Conclusions:**

RATS is safe and effective and took better short-outcomes than VATS in non-small cell lung cancer.

肺癌目前在全球癌症病例中约占15%，在癌症相关死亡病例中约占28%，是导致癌症相关死亡中最常见的类型，其发病率与死亡率在我国均已高居至癌症的首位^[1]^。虽然目前治疗肺癌的手段多种多样，但是对于早中期的非小细胞肺癌（non-small cell lung cancer, NSCLC）而言，外科手术是其公认的首选治疗方案，被认为是治疗早中期NSCLC的金标准^[2]^。近年来，随着微创外科技术的飞速进步以及快速康复外科（enhanced recovery after surgery, ERAS）的全面发展，微创外科手术已成为治疗NSCLC的的主流趋势。自1992年Lewis^[3]^首次报道应用电视胸腔镜手术（video-assisted thoracoscopic surgery, VATS）行肺叶切除术治疗肺癌以来，越来越多的研究^[4, 5]^证实：与传统开胸手术相比，VATS具有术后并发症发生率低、手术创伤小、术后疼痛轻、住院时间短等诸多优势。已被广大医师及患者所认可并应用于治疗各种胸外科疾病^[6]^。

2000年，一种全新的外科手术系统—达芬奇机器人手术（robot-assisted thoracic surgery, RATS）系统获得了美国食品药品监督管理局（Food and Drug Administration, FDA）批准并大量应用于临床各个领域，微创外科历史掀开了崭新的一页。然而，RATS治疗早中期NSCLC是否较VATS具有一定的优势仍存在广泛的争议^[7]^。因此，笔者对VATS与RATS肺癌根治术的术后中期治疗效果进行严格的病例配对对比研究，对目前胸外科学界广为关心的问题进行探讨分析。

## 1 资料与方法

### 1.1 一般资料

选取自2014年1月-2017年1月在沈阳军区总医院胸外科接受VATS肺癌根治术及RATS肺癌根治术的患者。纳入标准：术前经过各项常规化验及检查提示能够耐受肺叶切除术，无远处转移，术前未行放化疗，既往无胸部外伤史及胸部手术史，术后病理明确回报为非小细胞肺癌。病例配对条件：性别一致，年龄相仿（±5岁），肿瘤最大直径相近（±1 cm），肿瘤所在位置及术后TNM分期均相同。按照以上纳入标准及配对条件对RATS组及VATS组进行1:1配对，结果共有90例患者纳入研究，两组各45例，其中男性各24例，女性各21例。两组一般资料基本一致，差异无统计学意义（*P* > 0.05，表 1），具有可比性。

**1:** 2组患者一般临床资料比较（Mean±SD） The general clinical data were compared between the two groups (Mean±SD)

Index		RATS group	VATS group	*t*	*P*
*n*		45	45		
Gender	Male	24	24		
	Female	21	21		
Age (yr)		59.8±10.43	59.4±9.79	0.197	0.845
Tumor diameter (cm)		2.76±0.83	2.69±0.76	0.852	0.402
Tumor location	Left upper lobe	11	11		
	Left lower lobe	9	9		
	Right upper lobe	13	13		
	Right lower lobe	12	12		
TNM stage	Ⅰa	9	9		
	Ⅰb	8	8		
	Ⅱa	7	7		
	Ⅱb	11	11		
	Ⅲa	10	10		

### 1.2 手术方法

#### 1.2.1 胸腔镜组

患者取健侧卧位，双腔管气管插管，上胸部垫高，健侧单肺通气。采用腋下小切口法，于腋中线第7肋间或第8肋间切1.5 cm为进镜口并置入戳卡，于腋前线第4肋间小切口3 cm-5 cm为主操作口并使用一次性切口保护套将切口适当撑开，必要时适当延长该切口，视术中情况于腋后线第9肋间或肩胛下线第8肋间切1.5 cm作为辅助操作口。

#### 1.2.2 机器人组

患者取健侧卧位，头高脚低折刀位。双腔管气管插管，下胸部垫高，健侧单肺通气。采用三臂法，即1个镜头臂，2个器械臂，使用达芬奇机器人专用戳卡。于腋后线第8肋间切1.2 cm小口为进镜口，于肩胛下线第8肋间、腋前线与锁骨中线间第5肋间各切0.8 cm小口为器械操作口。并于腋中线第7肋间切3 cm-4 cm小口作为辅助操作口并使用一次性切口保护套对切口进行保护，助手通过此辅助切口使用相关器械与术者进行配合并最终由此切口取出手术标本^[8]^。

两组患者术中均先行病灶楔形切除，若病变靠近肺门无法行楔形切除则直接行肺叶切除，将标本送冰冻病理检查。若病理回报为恶性，则直接行标准肺叶切除+系统性淋巴结清扫。左侧清扫第2组-12组淋巴结，右侧清扫第2组-4组、7组-12组淋巴结。肺叶切除的具体解剖顺序按照刘伦旭等^[9]^报告的单向式或标准解剖式全胸腔镜肺叶切除术的术式标准进行。组织分离过程中随时用器械电凝止血。标本完整切除后均使用内镜取物器将其取出以防止其对胸腔及切口的污染。确切止血后逐层关胸，于进镜口常规留置一枚胸腔闭式引流管。术后拔除胸腔引流管的指征为：连续3日胸腔引流液的量低于150 mL、无乳糜液且咳嗽时无漏气。

### 1.3 统计学分析方法

采用SPSS 22.0统计软件包进行统计分析。计量资料均以（Mean±SD）表示，组间比较采用配对*t*检验，以*P* < 0.05为差异有统计学意义。

## 2 结果

### 2.1

两组患者术中相关资料比较两组的手术时间比较，差异无统计学意义（*P* > 0.05）。两组患者的术中失血量、淋巴结清扫总数以及淋巴结清扫站数比较：RATS组失血量明显少于VATS组，RATS组淋巴结清扫个数及站数均明显多于VATS组，差异均有统计学意义（*P* < 0.05，表 2）。

**2:** 两组患者术中相关资料比较（Mean±SD） The intraoperative clinical data were compared between the two groups (Mean±SD)

Group	RATS group	VATS group	*t*	*P*
Operation time (min)	184.52±39.51	50.30±32.33	-0.057	0.565
Intraoperative blood loss (mL)	50.30±32.33	208.60±132.63	8.009	0.000
Lymph node dissection	22.67±9.67	15.51±5.41	4.553	0.000
Lymph node dissection stations	6.31±1.43	4.91±1.04	5.250	0.000

### 2.2

两组患者术后相关资料比较两组的术后第1日胸引液的量比较：RATS组明显少于VATS组，差异具有统计学意义（*P* < 0.05）。两组的术后带管时间、术后住院时间以及术后胸引液总量比较，差异均无统计学意义（*P* > 0.05，表 3）。

**3:** 两组患者术后相关资料比较(Mean±SD) The postoperative clinical data were compared between the two groups (Mean±SD)

Group	RATS group	VATS group	*t*	*P*
Postoperative tube time (d)	10.55±5.31	11.45±4.22	-0.748	0.459
Postoperative hospital stay (d)	14.13±5.69	15.05±5.3	-0.675	0.504
Drainage of 1^st^ day (mL)	275.00±145.42	347.60±125.80	2.624	0.012
Total thoracic fluid (mL)	2, 159.75±1, 354.96	2, 739.75±1, 470.36	-1.684	0.100

## 3 讨论

肺癌预后较差，欧美地区报道的5年生存率为8%-16%，中国地区报道的5年生存率为6%-32%^[10]^。由于低剂量螺旋CT在肺癌筛查中的广泛应用，使得肺癌相关病死率降低了20%^[11]^。对于其中占有主导地位的非小细胞肺癌而言，外科手术仍然是其首选治疗方案。随着近些年肺癌疾病谱的变化、影像学的早期发现、手术器械的改进以及微创手术技巧的进步，多种样式的VATS技术已在国内外大量开展并取得了可观的突破性成果^[12]^。Cao等^[13]^进行了一项关于患者匹配性倾向评分的meta分析，结果表明，与常规开胸手术相比较，VATS显著降低了肺炎、持续性肺组织漏气、肾衰竭及房性心律失常等术后并发症的发生率。美国国家综合癌症网络（National Comprehensive Cancer Network, NCCN）已于2006年将VATS作为治疗早期肺癌的标准术式^[14]^。随着电视胸腔镜外科技术在临床中的广泛应用，人们也发现其在肺部手术中存在着一些有待解决的问题^[15, 16]^：二维平面术野缺乏一定的空间深度感觉，使得术者的手眼协调性有着不同程度的降低，在某些深部组织的操作中缺乏纵深视觉感受，在一定程度上增加了手术的风险；由于其对抗直觉的反向器械操作，延长了术者的学习曲线，术者需要大量的训练方能达到熟练水平；术中所使用的器械均无活动关节，仅有4个自由度，且无法除去人手操作的震颤，在某些狭小空间（如胸膜顶、膈肌角、肺动静脉段分支间等）行深部组织打结、缝扎、止血、分离及吻合等精细操作时难度增大；在手术初期，术者及助手长时间站立，且控制胸腔镜的助手需与术者密切配合方能使手术顺利进行，精力及体力均消耗过大，术者心理压力较大，在进行某些难度稍大的手术（如瘤体较大、与周围重要组织紧密粘连及全胸腔致密粘连等）时容易放弃全胸腔镜而改为中转开胸。以上因素均限制了微创胸部外科手术的进一步发展。随着三维成像技术、计算机图文技术、电脑全真模拟仿真技术以及机械自动化等工程技术的进步，由美国直觉外科公司（Intuitive Surgical Corporation）研发制造的达芬奇机器人手术系统（Da Vinci surgical system）应运而生并应用于临床，机器人的机械臂取代了外科医生的手臂，极致精准的微创外科时代诞生了^[17]^。

2002年，Melfi等^[18]^报道了全球首例应用达芬奇机器人手术系统行肺癌根治术。经过了多年大量临床实践的研究与验证，RATS行非小细胞肺癌根治术的安全性及可行性得到了广泛的肯定^[19, 20]^。我国于2006年由中国人民解放军总医院高长青院士首次引进达芬奇机器人手术系统并成功应用于心脏外科手术。截至2017年7月，我国大陆地区和香港地区共装机达芬奇机器人手术系统77台。其中中国香港地区培训使用1台，临床使用10台；中国大陆培训使用1台，临床使用65台（以上数据来源于机器人公司统计）。我院自2011年3月引进达芬奇机器人手术系统，并于2012年9月完成前期20例肺癌根治手术探索过程^[21]^。截至2017年11月，已完成各种达芬奇机器人普胸外科手术共1, 000余例。达芬奇机器人手术系统具有许多电视胸腔镜手术系统所不具备的优势^[22]^：由于其特殊的成像系统，可为术者提供突破人眼局限的高清晰3D手术视野并使其放大10倍-15倍，图像和控制手柄方向一致，其手眼协调性与传统开胸手术相似；术者自行控制镜头，无需助手扶镜；术者无需执行无菌操作规范而洗手上台站立操作，可直接坐在操控台旁，使术者疲劳感显著降低，可较为轻松地完成各种复杂手术；独特的Endo-Wrist仿真手腕具有7个自由度，突破人手仅有5个自由度的活动局限，且每秒可同步1, 300次，并完全滤除人手震颤，使得在行深部组织缝合打结以及在狭小空间行精细操作时更加灵活安全。近期Yang等^[23]^进行了一项关于达芬奇机器人手术与单孔胸腔镜手术治疗肺癌的匹配性倾向评分的对比研究，两组最终各纳入69例患者，其结果显示两组手术时间、术后住院天数及带管时间均无统计学差异，RATS组出血量明显少于VATS组[(80.84±46.49) mL *vs* (110.66±56.24) mL, *P*=0.037]，且RATS组清扫淋巴结的个数显著多于VATS组[(11.62±4.64) *vs* (11.05±5.80), *P*=0.014]。与本研究结论一致。李剑涛等^[24]^进行了一项单中心连续333例机器人辅助胸腔镜肺叶切除术治疗Ⅰ期非小细胞肺癌的临床资料回顾性分析，其研究结果证明：RATS肺叶切除术治疗Ⅰ期非小细胞肺癌安全有效，可很好地弥补传统胸腔镜手术的不足。充分证明了达芬奇机器人手术系统的上述优势。

本研究中纳入的90例患者术中均无大出血及输血，均无中转开胸及围术期死亡。两组患者的手术时间无统计学差异。也有相关文献[25, 26]报道RATS行肺癌根治术的手术时间较VATS长，不同术者所报道的手术时间也有所差别。笔者认为可能有如下几种原因：某些术者在刚开始开展此项技术时尚缺乏一定的经验，在学习曲线的初始阶段其手术时间必定较长；在进入胸腔内操作之前其机械臂系统的组装需要消耗一定的时间；由于不同术者所使用的技术及其手术习惯的不同，手术时间也必定有明显的差异。值得一提的是，较长的手术时间不等同于较差的术后结局。Agzarian等^[27]^进行的一篇*meta*分析表明：虽然达芬奇机器人手术系统行肺癌根治术的时间较电视胸腔镜手术系统行肺癌根治术的时间长，但是不会增加其中转率，且两者对患者术后整体功能影响的改善、住院时间及术后生存时间均无显著差异。

外科手术对患者所带来的创伤程度也是不容忽视的重要方面。术中失血量、术后胸引液的量以及术后疼痛都可以体现其创伤程度。本研究结果显示RATS组的术中失血量明显少于VATS组（*P*=0.000）且术后带管时间及术后胸引液的总量不多于VATS组。其可归功于如前所述的达芬奇机器人手术系统独特优势，由于其较高的精准性与灵活性，极大地减小了手术操作时其对血管等重要组织的损伤程度。Mungo等^[28]^进行的一项对比研究结果提示：在行肺癌根治术时，VATS更容易出现难以控制的术中大出血，而且其中转率也显著高于RATS。提示达芬奇机器人手术系统行肺癌根治术时更为安全可靠。张大坤等^[29]^进行了一项关于达芬奇机器人手术与胸腔镜辅助小切口手术对肺手术患者创伤程度的对比分析，该研究选取了40例达芬奇机器人手术的患者和48例胸腔镜辅助小切口手术的患者，运用了McGill疼痛问卷（McGill Pain Questionnaire, MPQ）调查法，分别对两组患者的术中出血量、术后疼痛程度、术后引流量、术后留置胸引管时间进行对比研究，结果提示达芬奇机器人手术能够明显减轻手术对患者造成的疼痛，同时能够减少术中及术后出血量以及术后带管时间。提示达芬奇机器人手术系统更加微创。

术中淋巴结清扫的彻底性是治疗非小细胞肺癌尤为关键的一步，对于明确分期、判断预后以及指导下一步治疗都有着不可忽视的作用，同时可以提高局部控制率并延长患者生存时间^[30]^。根据2006年欧洲胸科医生协会（European Society of Thoracic Surgeons, ESTS）发行的指南^[31]^指出：无论采取何种术式，针对可切除的非小细胞肺癌均应进行系统的淋巴结清扫。本研究结果提示RATS组术中淋巴结清扫个数及站数均显著多于VATS组[(22.67±9.67) *vs* (15.51±5.41), (6.31±1.43) *vs* (4.91±1.04), *P*=0.000]。近期David等^[32]^进行了一项大样本量的回顾性分析研究，共纳入了加州癌症登记中心2004年至2011年经手术治疗的Ⅰ期-Ⅲ期期NSCLC患者16, 393例，采用*Kaplan-Meier*和*Cox*风险比例模型进行数据研究，Ⅰ期患者经人口统计学和临床因素调整后淋巴结总数少于10枚者总生存率较大于或等于10枚者更低。结果显示：淋巴结样本总数为0、1-3、4-10的总生存率风险比分别为1.78（95%CI: 1.54-2.05, *P* < 0.000, 1）、1.43（95%CI: 1.27-1.59, *P* < 0.000, 1）、1.16（95%CI: 1.05-1.28, *P*=0.004）。其结果提示对于NSCLC患者而言，淋巴结样本总数影响其总体生存率和癌症相关生存率，但此结果与肿瘤分期有关系。因此术者应该行彻底的淋巴结清扫来最大限度地提高患者的生存率，从而使患者获益最大化。Alper Toker等^[33]^进行了一项关于常规开胸手术、VATS与RATS肺癌根治术淋巴结清扫效果的对比研究，该研究共纳入了270例患者，其研究结果表明：RATS组淋巴结清扫总数明显多于其他两组[(12.0±6.4) *vs* (11.7±4.7) *vs* (14.9±6.5), *P*=0.000, 7]，其中N1组淋巴结的清扫数量显著多于其他两组[(4.0±2.7）*vs* (3.8±2.1) *vs* (6.8±3.7), *P* < 0.000, 1]。刘星池等^[34]^进行了一项关于达芬奇机器人非小细胞肺癌根治术手术疗效的回顾性临床资料分析，共有202例患者纳入该研究，其结果表明：RATS组和VATS组的淋巴结清扫总数、淋巴结清扫站数、术后2年无进展生存率比较，RATS组均优于VATS组（*P* < 0.05）。充分体现了达芬奇机器人手术系统对淋巴结清扫的彻底性。笔者认为，由于达芬奇机器人手术系统具有高度清晰的成像系统以及比人手更为灵活精细的Endo-wrist仿真手腕，在清扫淋巴结时比传统胸腔镜更为方便安全。高度放大的3D手术视野几乎无任何死角，可以更加清晰地暴露肺门以及纵隔各区域的淋巴结及其组织，极大地减少了淋巴结残留的发生率。无论是在淋巴结的抓取游离，还是在避开淋巴结周围血管等方面，达芬奇机器人手术系统都有着无法超越的优势。基于达芬奇机器人手术系统精准微创的特质，未来必将会有越来越多的胸外科医师开展并推广此项技术。随着科学技术的进步，新一代的达芬奇机器人手术系统必然会使得手术的安全性、精准性以及可操作性进一步提升^[35]^。

为了减小偏倚，提高组间的均衡性，本研究采用了病例配对对比研究。本研究只对比了术后近期结果，若能扩大样本量，加之对比淋巴结清扫的具体情况及远期生存结果，将更能体现出机器人手术的独特优势。
